# Novel agents and clinical trials in castration-resistant prostate cancer: latest updates from 2023 ASCO-GU Cancers Symposium

**DOI:** 10.1186/s40164-023-00430-1

**Published:** 2023-08-01

**Authors:** Yuanhong Jiang, Siyu Wu, Rong Li, Jiazheng Yu, Jianyi Zheng, Zeyu Li, Mingyang Li, Kerong Xin, Zhenqun Xu, Shijie Li, Xiaonan Chen

**Affiliations:** Department of Urology, Shengjing Hospital of China Medical University, Shenyang, 110004 Liaoning People’s Republic of China

**Keywords:** CRPC, RDC, Amphiphiles and retargeted proteins, CAR-T, AKTi

## Abstract

Numerous novel and effective therapeutic agents and clinical trials addressing castration-resistant prostate cancer (CRPC) were reported during the 2023 American Society of Clinical Oncology-Genitourinary (ASCO-GU) Cancers Symposium. Notably, radionuclide drug conjugates (RDC), specifically 177Lu/111In-J591 and 225Ac-J591, exhibited enhanced therapeutic efficacy in treating patients with CRPC. Furthermore, promising treatment approaches for CRPC included dual anti-cytotoxic T-lymphocyte-associated protein 4 (CTLA-4) and anti-programmed death-1 (PD-1) blockade in rare tumors (DART)-Lorigerlimab, prostate stem cell antigen (PSCA)-directed chimeric antigen receptor (CAR)-T cell immunotherapy-BPX-601, and protein kinase inhibitor (AKTi)-CAPltello-280. We have summarized the latest CRPC treatment strategies presented at the 2023 ASCO-GU Cancers Symposium, along with recent advances in CRPC clinical trials.

## To the editor

Each year, the ASCO-GU Cancers Symposium showcases noteworthy developments and innovations in genitourinary oncology. We have comprehensively reviewed such notable advancements in drugs and novel therapies targeting CRPC, as presented at the 2023 ASCO-GU Cancers Symposium.

## RDC in prostate Cancer (PCa)

RDC drugs utilize antibodies or small molecules to modulate specific targets and deliver cytotoxic or imaging agents to the target location, resulting in localized radiation from the radioisotope on the target tissue for efficient and precise treatment while minimizing systemic exposure and radiation-induced toxicity to other tissues [1].

In a randomized, double-blinded phase II study, radioactive 177Lu and 111In, combined with ketoconazole or hydrocortisone, was used to label the anti-prostate-specific membrane antigen (PSMA) monoclonal J591 (NCT00859781). The results indicated a significant reduction in prostate-specific antigen (PSA) levels in most patients with non-metastatic CRPC (M0CRPC) treated with radiolabeled J591 and ketone/HC (PSA decline ratio > 50% (PSA50): 82% and 71% in the177Lu and 111In groups, respectively; PSA decline ratio > 90% (PSA90): 50% and 35% in the 177Lu and 111In groups, respectively). Biochemical progression-free survival (bPFS) was 18.67 and 8.87 months in the 177Lu and 111In groups, respectively, with a significantly higher 18-month PFS in the 177Lu group; however, hematologic toxicity was more common in this group [2]. These findings support the development of anti-PSMA radioimmunotherapy for locally advanced PCa; however, the optimal radionuclide and targeting agent remain to be determined.

Another new triple therapy involving 225Ac-J591 (a PSMA-targeted radionuclide therapy), pembrolizumab, and an androgen receptor pathway inhibitor (ARPI) demonstrated a significant PSA response in both phase I/II trials (NCT04946370). After six months of follow-up, 33% (4/12) of the patients remained progression-free. However, 58% (7/12) of the patients developed unexpected cytokine release syndrome (CRS) 7–14 days after treatment, characterized by a morbid rash, fever, and low blood cell count. Nevertheless, patient responses typically improved within one week after discontinuing ARPI. Additionally, typical immune-related adverse events (irAEs) occurred in 33% (4/12) of the patients, all of which were manageable [3].

**Table 1:** Basic information on novel agents for CRPC patients from ASCO-GU 2023

Classification	Structure	Mechanism	Side effects	Reference
RDC	utilizes radionuclides energetically chelated to small molecules or monoclonal antibodies designed to target antigens	target and deliver a precise amount of cytotoxic radiation to prostate cancer cells while sparing the surrounding normal tissues	Neutropenia, thrombocytopenia, abdominal pain, increased ALT levels. diarrhea, thrombocytopenia, neutropenia, nausea, fatigue, xerostomia, AST	[1]
DART	combine variable domains of two antigen-binding segments linked to two independent poly-peptide linkers. Each variable domain is constructed by associating one light-chain and one heavy-chain covalently linked using disulfide bridges.	Recruit and redirect immune effector cells to kill tumor cells or block various signaling pathways by inhibiting either the ligand or the receptor, redirect effector cells against cancer cell targets in a major histocompatibility complex-independent manner, thereby avoiding immune escape strategies of MHC downregulation by cancer cells.	CRS, organ dysfunction, neurotoxicity	[4]
CAR-T	Autologous T cells genetically modified to contain a specific CAR and an inducible co-stimulatory domain.	CAR-T cells can recognize tumor antigens in an HLA-independent manner, binding to target proteins on tumor surfaces, promoting T cell proliferation and cytokine secretion, as well as the secretion of anti-apoptotic proteins.	On-target/off-tumor toxicity: binding of CAR-T cells to target antigens expressed on normal cells On-target/on-tumor toxicity: CRS	[6]
AKTi	pyrimidine compounds and alkyl phospholipids	ATP-Competitive AKTi blocks downstream signaling pathways by trapping Akt in a phosphorylated but nonfunctional state, thus inhibiting tumor cell growth, survival, proliferation, and apoptosis. Allosteric AKTi maintains Akt in a locked conformation, blocking the association of Akt and PIP3 at the membrane level, leading to inhibition of Akt activation.	Side effects such as diarrhea, fatigue, nausea, and rash.	[8]

AEs: Adverse events, AKTi: Protein kinase inhibitor, ALT: Alanine aminotransferase, AST: Acute septic thyroiditis, ATP: Adenosine triphosphate, CAR: Chimeric antigen receptor, CAR-T: Chimeric antigen receptor-T cell,

CRPC: castration-resistant prostate cancer, CRS: cytokine release syndrome, DART: Dual antibody blockade in rare tumors, irAEs: Immune-related adverse events, RDC: Radionuclide drug conjugates

**Table 2:** Outcomes of novel agents and clinical trials in CRPC from ASCO-GU 2023

Dosing Regimens	Indication	Classification	Intervention	Regimen Backbone	Patient number	OS	PFS	ORR	PSA50	Clinical trail number	Reference
177Lu-J591	M0CRPC	RDC	177Lu-J591 + Keto + HC vs. 11In-J591(placebo) + Keto + HC	Keto + HC	55	-	18.67mon	-	82% (PSA90:50%)	NCT00859781	[2]
225Ac-J591	mCRPC	RDC	225Ac-J591 +Pemb + ARPI vs. Pemb + ARPI	Pemb + ARPI	76	-	33% > 6 mon	-	50%	NCT04946370	[3]
Lorigerlimab	mCRPC	DART	-	-	42	-	-	25.7%	28.6% (PSA90:21.4%)	NCT03761017	[5]
BPX-601	mCRPC	CAR-T	-	AR antagonist + Taxane	151	-	-	14.3%	42.9%	NCT02744287	[7]
Capivasertib	mCRPC	AKTi	Capivasertib + docetaxel vs. placebo + docetaxel	Docetaxel	790	31.5mon	7.03 mon	-	45%	NCT05348577	[9]

AKTi: Protein kinase inhibitor, AR: Androgen receptor, ARPI: Androgen receptor pathway inhibitor, CAR-T: Chimeric antigen receptor-T cell, DART: Dual antibody blockade in rare tumors, HC: hydrocortisone, Keto: ketoconazole,

mCRPC: Metastatic castration resistant prostate cancer, M0CRPC: non-metastatic castration resistant prostate cancer, mon: month, ORR: Objective response rate, OS: overall survival, PSA: prostate specific antigen,

PSA50: PSA decline ratio > 50%, PSA90: PSA decline ratio > 90%, PFS: progression free survival, Pemb: Pembrolizumab, RDC: Radionuclide drug conjugates

## Novel regimens in PCa

A study reported data on lorigerlimab (a DART molecule [4] that enhances CTLA-4 blockade of dual expression while maintaining a maximal blockade of PD-1) in a trial of 42 PSA-assessable patients with mCRPC (35 RECIST-assessable), with an objective response rate (ORR) of 25.7% (9/35). Only four cases were discontinued owing to unrelated fatal adverse events. Lorigerlimab demonstrated a manageable safety profile with encouraging anti-tumor activity in patients with chemorefractory mCRPC (NCT03761017) [5]. Another multicenter trial presented preliminary results of a phase 1 multicenter trial on BPX-601, an autologous PSCA-directed CAR-T cell immunotherapy [6] that enhances T cell potency and persistence by expressing a mature-induced MyD88/CD40 costimulation switch (NCT02744287). A PSA50 response was observed in 42.9% (3/7) of the patients on day 28. Preliminary results based on RECIST indicated a partial response (PR) of 14.3% (1/7) and stable disease of 42.9% (3/7). Disease progression occurred in only 14.3% (1/7) of the patients, and 14.3% (1/7) of the patients maintained stable disease (SD) for > 9 months [7].

In a phase III study, the efficacy of AKTi-CAPltello-280 (effective selective inhibition [8] of AKT1/2/3) in combination with docetaxel was evaluated, and an increase in overall survival (OS) was observed in patients with mCRPC. Although the Phase III trial is ongoing (NCT05348577), the results from the Phase II trial revealed that patients achieved a median OS of 31.5 months, clinical PFS of 7.03 months, and PSA50 rate of 45% (NCT05348577) [9].

Overall, the 2023 ASCO-GU Cancer Symposium showcased significant advancements in the therapeutic area of CRPC, as evidenced by the findings presented in Tables 1 and 2. The symposium highlighted the emergence of many encouraging new drugs and clinical trials, creating the potential for novel treatment strategies for CRPC.

## Data Availability

The material supporting the conclusion of this study has been included in the article.

## Abbreviations

ALT: Alanine aminotransferase
AST: Acute septic thyroiditis
ADT: Androgen deprivation therapy
AEs: Adverse events
AKTi: Protein kinase inhibitor
AR: Androgen receptor
ARPI: Androgen receptor pathway inhibitor
ATP: Adenosine triphosphate
bPFS: Biochemical progression-free survival
CAR: Chimeric antigen receptor
CAR-T: Chimeric antigen receptor-T cell
CRPC: Castration resistant prostate cancer
CRS: Cytokine release syndrome
CTLA-4: Cytotoxic T-lymphocyte-associated protein 4
DART: Dual anti-CTLA-4 & anti-PD-1 blockade in rare tumors
HC: Hydrocortisone
irAEs: Immune-related adverse events
Keto: Ketoconazole
M0CRPC: Non-metastatic castration resistant prostate cancer
mCRPC: Metastatic castration-resistant prostate cancer
mon: month
ORR: Objective response rate
OS: Overall survival
PCa: Prostate cancer
PD-1: Programmed death-1
Pemb: Pembrolizumab
PFS: Progression-free survival
PSA: Prostate specific antigen
PSA50: PSA decline ratio > 50%
PSA90: PSA decline ratio > 90%
PSCA: Prostate stem cell antigen
PSMA: Prostate-specific membrane antigen
RDC: Radionuclide drug conjugates
SD: Stable disease
